# Supplementary material for: A Mechanochemical Route for ZnS Nanocrystals, and Batch Sorting along Size Distribution
Source: Nanomaterials (Basel). 2019 Sep 15;9(9):1325. doi: 10.3390/nano9091325 (PMC6781050; doi:10.3390/nano9091325)
Supplement: Supplementary file 1 [file nanomaterials-09-01325-s001.pdf]

Article

A Mechanochemical Route for ZnS Nanocrystals, and Batch Sorting Along Size Distribution

Pengfei Hu, Chen Xie, Zhihui Mao and Xue Liang *

Laboratory for Microstructures, Shanghai University, Shanghai 200444, P. R. China;
hpf-hqx@shu.edu.cn (P.F.H.); FHYKong@shu.edu.cn (C.X.); maozhihui@shu.edu.cn (Z.H.M.)

* Correspondence: liangxue@shu.edu.cn (X.L.); Tel.:+86-21-66135030 (X.L.)

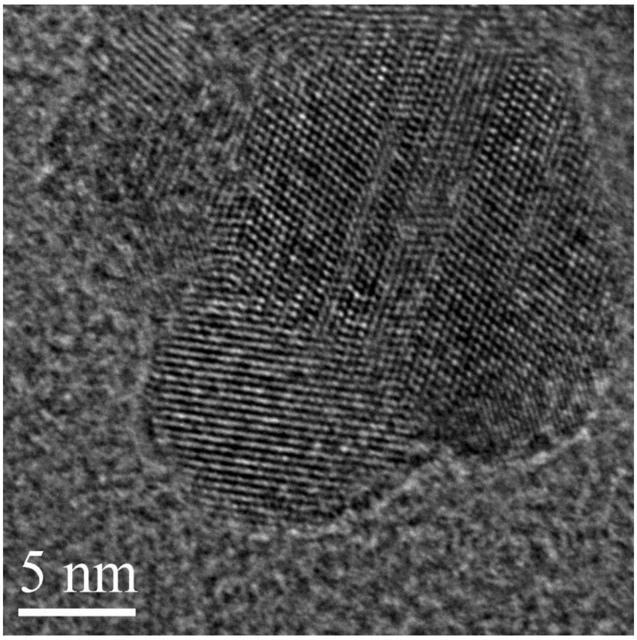

Figure S1. HRTEM images of Q-ZnS-c nanocrystals, showing twinning structures and agglomeration behavior.

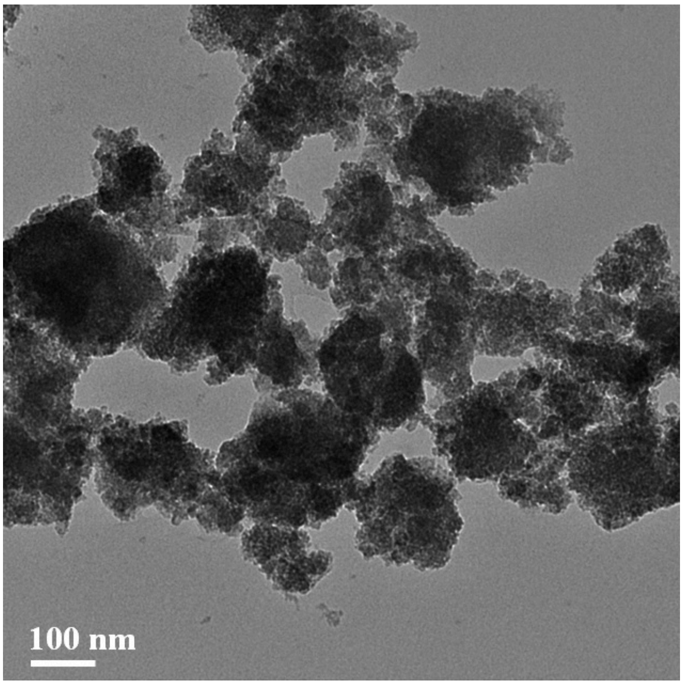

Figure S2. TEM images of ZnS samples synthesized through mechanochemical route without SDBS, indicating the large aggregates of nanoparticles.
